# Post-mastectomy Breast Reconstruction: Experience From the National Cancer Institute of Panama

**DOI:** 10.7759/cureus.96563

**Published:** 2025-11-11

**Authors:** Ricardo Gollini, Moisés Cukier, Blanca Armas, María Paula Paredes, Héctor Martínez, José M. Samudio

**Affiliations:** 1 Clinical Research, Cevaxin, Panama City, PAN; 2 Surgery, Pacifica Salud Hospital, Panama City, PAN; 3 Medical Education and Research, Caja del Seguro Social, Panama City, PAN; 4 Plastic and Reconstructive Surgery, National Cancer Institute, Panama City, PAN

**Keywords:** breast reconstruction, complication, flaps, implants, mastectomy

## Abstract

Introduction

Breast cancer is the most prevalent cancer among women worldwide and in Panama, where it represents a significant public health concern. Advances in early diagnosis and treatment have improved survival rates, shifting attention toward post-treatment quality of life. Mastectomy, while essential for curative treatment, often leads to physical and psychological challenges. Breast reconstruction has emerged as a key strategy to address these effects, with techniques varying between implant-based and autologous approaches, and timing options including immediate or delayed reconstruction. Despite its benefits, access to reconstruction remains limited in many countries. At the National Cancer Institute (NCI) of Panama, which serves the majority of breast cancer patients nationwide, there is limited documentation of post-mastectomy reconstruction practices. This study aims to describe the experience of breast reconstruction following total mastectomy at NCI between 2015 and 2019, providing insights into institutional practices and contributing to future strategies for equitable post-mastectomy care.

Materials and methods

A descriptive, observational, and retrospective study was conducted at the National Cancer Institute (NCI) of Panama. The study included female patients over 18 years of age who were diagnosed with breast cancer and underwent total mastectomy followed by immediate or delayed breast reconstruction between 2015 and 2019. Surgical techniques evaluated included implant-based reconstruction, latissimus dorsi flap with or without implant, and transverse rectus abdominis myocutaneous (TRAM) flap. Patients who underwent oncoplastic procedures after breast-conserving surgery were excluded. Data collected encompassed sociodemographic, clinical, and surgical variables, as well as comorbidities and postoperative complications. Data were extracted from electronic medical records and analyzed using Epi Info 7.2.5.

Results

A total of 185 breast reconstructions were recorded, representing 13% of the total mastectomies performed. The mean age was 48 ± 9 years. According to the timing of reconstruction, 72% were delayed and 28% immediate. The mean time between mastectomy and delayed reconstruction was 3.2 years. The main reconstruction technique was tissue expander/prosthesis placement (55%) followed by latissimus dorsi flap reconstruction (26%). Early and late complications were observed in 22.7% and 17.3% of cases, respectively, with cellulitis being the most frequent complication in both periods.

Conclusions

At the NCI of Panama, an average of 37 breast reconstructions are performed annually in patients who have undergone total mastectomy. Most of these procedures are delayed using implant or tissue expander, with an average interval of 3.2 years between mastectomy and reconstruction.

## Introduction

Breast cancer represents approximately 12.5% of all new annual cancer cases worldwide, making it the most common malignancy among women. It is estimated that 1 in 8 women will be diagnosed with breast cancer during their lifetime [1]. In the United States alone, 310,720 new cases are expected in 2024 [2]. In Panama, breast cancer is the leading cause of cancer in women, with an incidence rate of 63.7 cases per 100,000 inhabitants and approximately 1,000 cases treated annually at the National Cancer Institute (NCI) [3].

Advances in early diagnosis and continuous improvements in treatment have significantly increased survival rates in recent years [4,5]. This progress has shifted attention toward strategies that enhance the quality of life for patients after cancer treatment.

Mastectomy remains a cornerstone of curative management for breast cancer [5]. However, it often entails physical and psychological consequences, impacting self-esteem, femininity, and intimacy [6]. To address these challenges, post-mastectomy breast reconstruction has become an essential component of comprehensive care for breast cancer survivors.

Breast reconstruction can be performed using implant-based techniques or autologous tissue, with the choice influenced by patient characteristics, oncologic considerations, and clinical context [7-9]. Implant-based reconstruction offers shorter recovery and avoids donor site morbidity [9], while autologous reconstruction, using flaps such as TRAM, latissimus dorsi, or DIEP, provides a more natural appearance and avoids prosthetic materials [8,9].

The timing of reconstruction may be immediate (at mastectomy) or delayed, depending on surgical extent, adjuvant treatments, comorbidities, and patient preferences [10]. Despite proven benefits, reconstruction rates remain low and variable worldwide, reflecting disparities in access and resource allocation [10,11].

In Panama, the NCI is the main public hospital specialized in comprehensive oncologic care, serving most breast cancer patients nationwide. However, national data on post-mastectomy reconstruction are scarce, limiting understanding of local practices and outcomes. Therefore, this study aims to describe the experience of breast reconstruction following total mastectomy at the National Cancer Institute of Panama between 2015 and 2019.

This article was previously presented as a poster at the 2024 Cancer Panama Congress on March 14, 2024.

## Materials and methods

A descriptive, observational, and retrospective study was conducted at the NCI of Panama, focusing on patients diagnosed with breast cancer who underwent total mastectomy followed by breast reconstruction between 2015 and 2019.

The study included female patients over 18 years of age with a histologically confirmed diagnosis of breast cancer who underwent either immediate or delayed breast reconstruction following total mastectomy. The surgical techniques evaluated included expander/implant-based reconstruction, latissimus dorsi flap reconstruction with or without implant/expander, and TRAM flap reconstruction. Patients who underwent oncoplastic procedures following breast-conserving surgery were excluded from the analysis.

All patients during the study period were included, meaning the sample represented the entire population. The list of eligible patients was obtained from the NCI medical records department, which conducted a database search in the surgical and pathology registries using the specific procedure codes evaluated in this study. Data were extracted from both outpatient and inpatient electronic medical records using a standardized data abstraction form. Prior to data collection, the investigators received training from specialists in Plastic Surgery and Surgical Oncology to ensure accuracy and consistency. The data abstraction form was also evaluated by external peers who were not part of the research team to ensure its validity and comprehensiveness. In case of discrepancies, the records were jointly reviewed until consensus was reached.

Data collection encompassed sociodemographic variables, clinical characteristics related to breast cancer (such as histological type and oncologic treatment received), and surgical variables including the type of mastectomy, reconstruction technique and timing, and the presence of early or late postoperative complications. Additionally, comorbidities such as smoking and alcohol consumption, diabetes mellitus, hypertension, and obesity were recorded. Missing or incomplete data were coded as "not available" and excluded from specific analyses but retained in the dataset for overall descriptive purposes.

The data were tabulated in Microsoft Excel (Office 365) and analyzed with Epi Info version 7.2.5. Categorical variables were summarized as frequencies and percentages, while continuous variables were described using means and standard deviations.

The study is duly registered in the Clinical Research Registry System of the Ministry of Health of Panama (RESEGIS) under the code 2033. It received approval from the Institutional Ethics Research Board of the University of Panama, as well as authorization from the NCI for access to clinical records. Given the minimal risk associated with the study design, the ethics committee waived the requirement for informed consent. Patient confidentiality was strictly maintained by excluding any personally identifiable information from the dataset.

## Results

**Figure 1:**
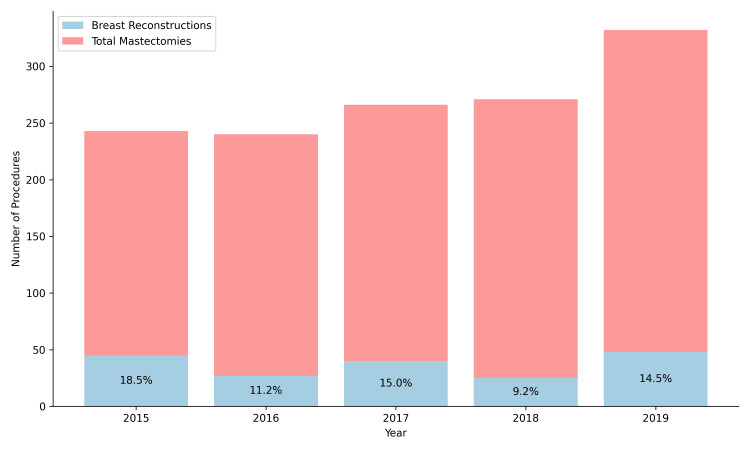
Proportions of breast reconstruction and total mastectomies per year at NCI, 2015-2019. NCI: National Cancer Institute.

During the study period, a total of 1,350 mastectomies and 185 post-mastectomy breast reconstructions were performed. This corresponds to an average of 37 reconstructions per year, representing approximately 13.7% of all mastectomies conducted during the study period. The annual distribution of mastectomies and reconstructions is presented in Figure 1.

The mean age of the total population was 48±9 years. In the group that underwent immediate breast reconstruction, the mean age was 44.8±9 years, while in the group that underwent delayed reconstruction, it was 49.5±9 years. The rest of the patient characteristics are described in Table 1.

**Table 1:** Clinical characteristics of patients with breast reconstruction at NCI, 2015-2019. NCI: National Cancer Institute.

Characteristics	Value, n	Value, %	95% CI
Mean age, years	48 (9)	-	-
Years, range	18-76	-	-
Smoking			
Yes	9	4.86	2.25-9.03
No	176	95.14	90.97-97.75
Arterial hypertension			
Yes	46	75.14	68.26-81.18
No	139	24.86	18.82-31.74
Diabetes mellitus			
Yes	19	10.27	6.30-15.57
No	166	89.73	84.43-93.70
Body mass index			
Underweight	1	0.54	0.01-2.97
Normal weight	46	24.86	18.82-31.74
Overweight	47	25.41	19.30-32.31
Obesity	65	35.14	28.27-42.48
Undetermined	26	14.05	9.39-19.91
Neoadjuvant chemotherapy			
Yes	80	43.24	35.99-50.71
No	105	56.76	49.29-64.01
Post-operative radiotherapy			
Yes	132	71.35	64.26-77.75
No	53	26.65	22.25-35.74
Stage			
I	16	8.65	5.02-5.66
II	80	43.24	35.99-50.71
III	54	29.19	22.75-36.31
IV	2	1.08	0.13-3.85
Undetermined	33	17.84	12.61-24.13

**Figure 2:**
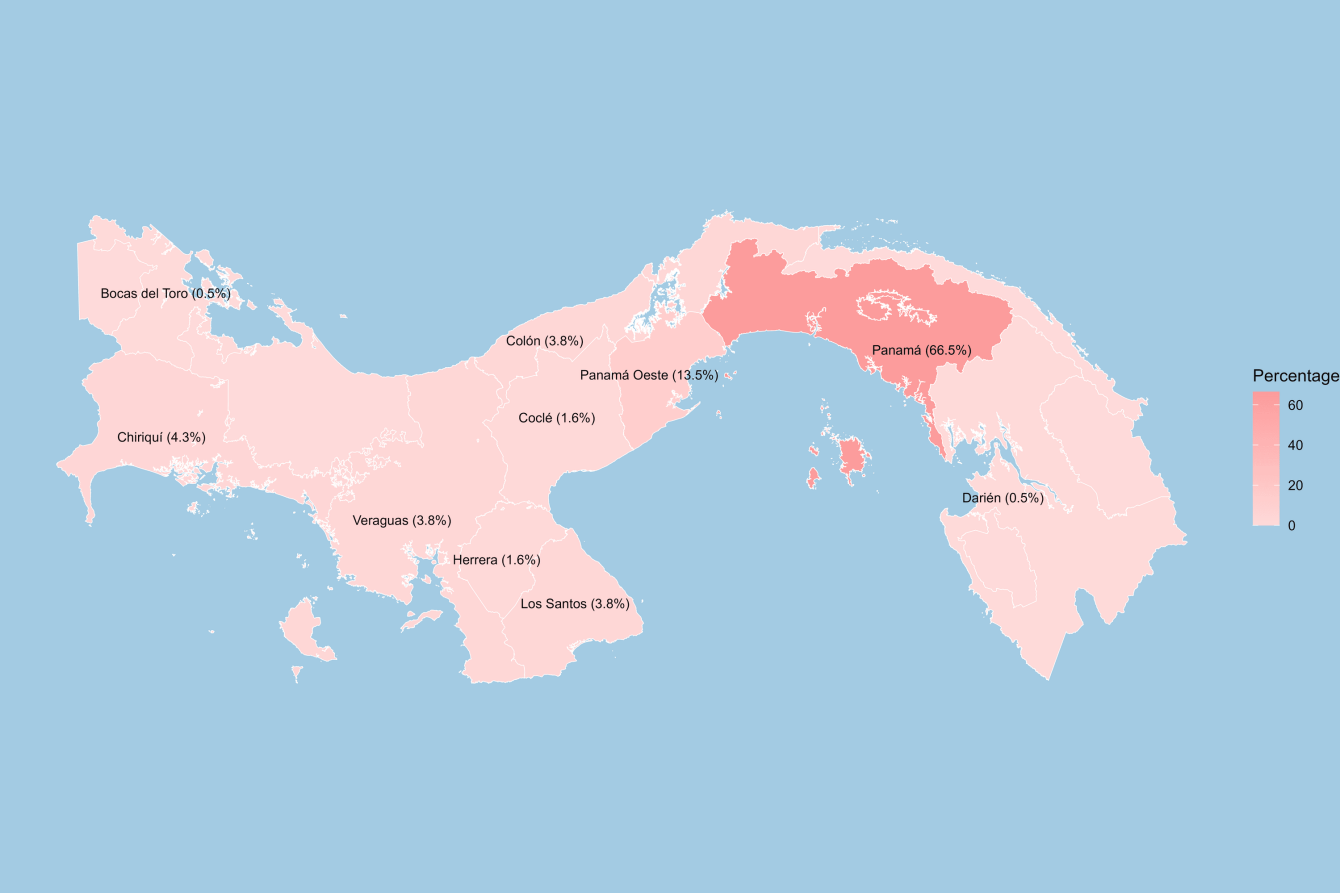
Geographical distribution of the of patients with breast reconstruction at NCI, 2015-2019. NCI: National Cancer Institute.

Most of the patients were originally from Panama, with 123 (66.5%), followed by Panama Oeste with 25 (13.5%) and Chiriquí with 8 (4.3%). The geographic distribution of patient residence is illustrated in Figure 2.

The histological type of breast cancer was determined according to the histopathological report performed at the NCI. Infiltrating ductal carcinoma was reported in 80.5% (149) of cases followed by ductal carcinoma in situ with 6.5% (12). The remaining histological types are summarized in Table 2.

**Table 2:** Histological type of breast cancer in patients with breast reconstruction at NCI, 2015-2019. NCI: National Cancer Institute.

Histology	Value, n	Value, %	95% CI
Invasive ductal carcinoma	149	80.54	74.09-85.98
Ductal carcinoma in situ	12	6.49	3.40-11.06
Invasive lobular carcinoma	10	5.41	2.62-9.72
Lobular carcinoma in situ	4	2.16	0.56-5.44
Phyllodes tumor	6	3.24	1.20-6.93
Others	4	2.16	0.56-5.44
Total	185	100.00	

Most breast reconstructions performed during the study period were delayed (72%), with an average interval of 3.2 years between mastectomy and reconstruction. The average hospital stay was 3.9 days (SD ±8.2) for immediate reconstructions and 3.7 days (SD ±4.1) for delayed reconstructions. Regarding the type of mastectomy performed, the majority of patients underwent a modified radical mastectomy (146, 78.9%), while the remaining received a total simple mastectomy (39, 21.1%).

In terms of breast reconstruction techniques, implant/expander placement was the most commonly used, with 102 (55.1%) cases. The remaining reconstructions were performed using autologous techniques: latissimus dorsi flap in 49 (26.5%) cases and TRAM flap in 34 (18.4%) cases. The distribution of breast reconstruction techniques according to the timing of reconstruction is shown in Table 3.

**Table 3:** Breast reconstruction techniques according to the timing of reconstruction at the NCI, 2015-2019. NCI: National Cancer Institute; TRAM: transverse rectus abdominis myocutaneous.

Breast reconstruction techniques		Immediate			Delayed		
		Value, n		Value, %	Value, n		Value, %
Implant/expander		47		92.16	55		41.04
Latissimus dorsi flap		2		3.92	47		35.07
TRAM flap		2		3.92	32		23.88
Total		51		100.00	134		100.00

Among the patients included in the study, 132 (71.4%) received postoperative radiation therapy. Within this subgroup, 107 patients (81.1%) underwent delayed breast reconstruction, while the remaining received immediate reconstruction. Among those who underwent delayed reconstruction, the most commonly employed techniques were the latissimus dorsi flap in 42 patients (39.3%), implant or tissue expander placement in 37 patients (34.6%), and TRAM flap in 28 patients (26.2%).

Complications were recorded based on whether they occurred early (≤30 days) or late (>30 days). Early and late complications were observed in 22.7% (n=42) and 17.3% (n=32) of cases, respectively. When analyzed by the timing of reconstruction, patients who underwent immediate reconstruction (n=51) experienced early complications in 31.4% of cases (n=16) and late complications in 19.6% (n=10). In contrast, among those who received delayed reconstruction (n=134), early complications occurred in 19.4% of cases (n=26) and late complications in 16.4% (n=22). Early and late complications in patients who underwent immediate and delayed breast reconstruction are presented in Tables 4, 5.

**Table 4:** Early and late complications in patients with immediate breast reconstruction at NCI, 2015-2019. NCI: National Cancer Institute.

Complications	Early		Late	
	Value, n	Value, %	Value, n	Value, %
Cellulitis	10	62.50	4	40.00
Seroma	3	18.75	1	10.00
Dehiscence	-	-	4	40.00
Epidermolysis	2	12.50	-	-
Expander damage	-	-	1	10.00
Fat necrosis	-	-	-	-
Flap suffering	1	6.25	-	-
Hematoma	-	-	-	-
Total	16	100.00	10	100.00

**Table 5:** Early and late complications in patients with delayed breast reconstruction at NCI, 2015-2019. NCI: National Cancer Institute.

Complications	Early		Late	
	Value, n	Value, %	Value, n	Value, %
Cellulitis	9	34.62	9	40.91
Seroma	9	34.62	2	9.09
Dehiscence	4	15.38	2	9.09
Epidermolysis	1	3.85	-	-
Expander damage	-	-	5	22.73
Fat necrosis	1	3.85	4	18.18
Flap suffering	1	3.85	-	-
Hematoma	1	3.85	-	-
Total	26	100.00	22	100.00

According to the reconstructive technique, the technique with the highest percentage of early complications was breast reconstruction using a latissimus dorsi flap (49, 26.53%), while, in late complications, it was the technique based on an expander/implant (38, 20.59%). Table 6 shows the complication percentages according to the reconstructive technique.

**Table 6:** Complications according to breast reconstruction technique at NCI, 2015-2019. NCI: National Cancer Institute; TRAM: transverse rectus abdominis myocutaneous.

Breast reconstruction techniques	Early		Late	
	Value, n	Value, %	Value, n	Value, %
Implant/expander	22	52.38	21	65.62
Latissimus dorsi flap	13	30.95	6	18.75
TRAM flap	7	16.67	5	15.63
Total	42	100	32	100

## Discussion

Breast reconstruction after total mastectomy has become an integral component of comprehensive breast cancer care, improving psychosocial well-being and quality of life for survivors. Globally, access to reconstruction remains highly variable, influenced by patient age, comorbidities, insurance coverage, adjuvant therapy, availability of specialized resources, and patient preference, with reported rates ranging from 5% to 60% [10,11].

In our study, 1,350 mastectomies and 185 reconstructions were performed between 2015 and 2019 at the NCI, yielding a reconstruction rate of 13.7%. This relatively low rate may reflect institutional constraints, such as limited operating room availability. For example, in 2019, only 315 of 4,015 surgical procedures at the NCI were performed by the Plastic Surgery Division [12].

Both immediate breast reconstruction (IBR) and delayed breast reconstruction (DBR) offer distinct benefits and challenges. IBR provides psychological advantages and reduces the total number of surgeries, often optimizing cosmetic outcomes [13]. However, in patients requiring post-mastectomy radiotherapy (PMRT), IBR may increase complication risks such as capsular contracture, wound dehiscence, and implant loss and can interfere with the timely initiation of radiotherapy [14,15]. These complications occur because irradiated tissue exhibits impaired healing, fibrosis, and increased susceptibility to infection, which compromise both aesthetic results and oncologic safety [16,17]. For this reason, DBR is generally recommended when PMRT is anticipated, as it allows completion of oncologic treatment before reconstruction, reducing the risk of adverse outcomes and preserving reconstructive options [18,19]. In our cohort, 71.4% of patients received postoperative radiotherapy, and stages II and III accounted for 72% of cases, likely contributing to the predominance of DBR (72%). While DBR is commonly chosen under these circumstances, it is important to emphasize that multiple strategies exist to integrate oncologic treatment and reconstruction, including tissue expander “bridge” pathways, immediate implant-based or autologous reconstructions, and hybrid approaches [20,21]. The decision should be individualized within a multidisciplinary framework, balancing oncologic priorities, patient characteristics, and preferences.

The recommended interval for DBR varies according to clinical context. Most guidelines suggest performing reconstruction 6-12 months after completion of adjuvant therapy, allowing adequate tissue recovery and reducing complication risk [21]. However, this period can be extended for patients with high-risk features such as obesity, advanced stage, or those requiring radiotherapy, where fibrosis and wound healing concerns may necessitate longer delays [18,21]. In our cohort, the average interval was approximately three years, which exceeds typical recommendations and likely reflects both patient complexity and institutional constraints, including limited operating room availability. These findings underscore the need for strategies to shorten delays, such as optimized scheduling and multidisciplinary coordination.

Regarding technique selection, implant-based reconstruction was the most common overall (55.1%), particularly in immediate cases (92%), while autologous techniques (latissimus dorsi and TRAM flaps) predominated in delayed reconstructions. Each approach carries specific advantages and limitations: implant-based reconstruction offers shorter operative time and recovery but is associated with complications such as infection and capsular contracture [22,23]. Autologous reconstruction provides superior aesthetic and psychosocial outcomes but involves longer, more complex surgeries and higher systemic complication risks [24-26]. Complication rates in our cohort were 22.7% (early) and 17.3% (late), consistent with published ranges [27,28]. These outcomes highlight the need for prospective studies to identify predictors of complications and optimize patient selection.

Limitations

This study is limited by its retrospective design and reliance on electronic medical records, which may contain incomplete or non-standardized data. Additionally, patients undergoing oncoplastic procedures after breast-conserving surgery were excluded, as these cases do not involve the Plastic Surgery Division, limiting generalizability. Despite these limitations, this study provides the first institutional overview of post-mastectomy breast reconstruction in Panama, offering valuable insights into surgical practices and access to care. Future research should focus on long-term outcomes and patient-reported measures to inform national policy development.

## Conclusions

This study provides the first institutional overview of post-mastectomy breast reconstruction practices at the National Cancer Institute of Panama between 2015 and 2019. During this period, 1,350 mastectomies and 185 reconstructions were performed, representing a reconstruction rate of 13.7%. Most procedures (72%) were delayed, with an average interval of 3.2 years, and implant-based techniques predominated (55.1%). Early complications occurred in 22.7% of cases, with cellulitis being the most frequent.

These findings underscore the need to improve access to timely and safe reconstructive options and to develop national strategies that promote equitable and comprehensive post-mastectomy care. Future research should explore barriers to reconstruction and evaluate long-term outcomes, including patient satisfaction, aesthetic results, and quality of life. Prospective multicenter studies with standardized complication reporting are essential to identify risk factors and optimize outcomes. Additionally, incorporating data on breast-conserving and oncoplastic procedures will provide a broader perspective on breast cancer treatment in Panama.
